# Patients with Parkinson Disease in a Traditional Korean Medicine Hospital: A Five-Year Audit

**DOI:** 10.1155/2021/6842863

**Published:** 2021-01-12

**Authors:** Jung Yun Yang, Chul Jin, JiEun Lee, Han-gyul Lee, Seung-Yeon Cho, Seong-Uk Park, Woo-Sang Jung, Sang-Kwan Moon, Jung-Mi Park, Chang-Nam Ko, Ki-Ho Cho, Seungwon Kwon

**Affiliations:** ^1^Department of Cardiology and Neurology, College of Korean Medicine, Kyung Hee University, Seoul 02447, Republic of Korea; ^2^Department of Korean Medicine Cardiology and Neurology, Graduate School, Kyung Hee University, Seoul 02447, Republic of Korea

## Abstract

Parkinson disease (PD) patients' demand for complementary and alternative medicine (CAM) has thus been increasing. We investigated the characteristics of PD patients who visited the Korean Medicine Hospital, the official CAM clinic in Korea. The medical records of PD patients were reviewed retrospectively. The demographic and disease-related characteristics, motivation for visiting, and treatment modalities were collected and analyzed. Medical records from 618 patients were reviewed. Most patients (67.6%) had been already diagnosed with PD at the initial visit. The most frequent complaint was gait disturbance. Previously diagnosed patients visited most frequently for add-on CAM therapies. The most frequently used CAM therapy was acupuncture. The most frequently prescribed herbal medicine was modified Ukgansan. We found the main reason for seeking out CAM was to compensate for the effects of conventional therapies. Further prospective studies will be necessary to collect enough data and evaluate the effectiveness of CAM therapies.

## 1. Introduction

There is a growing demand for complementary and alternative medicine (CAM) therapies to counter the limitations [1–4] in various conventional treatments of Parkinson disease (PD). Korea and other Far Eastern Asian countries have traditional East Asian medicine that has been used for thousands of years [5]. Therapies used in traditional medicine such as acupuncture, moxibustion, and herbal medicine are largely included in CAM therapies and are still actively used for the health care in each country. In particular, Korea has a unique dualized medical system. There are medical doctors who perform western medicine and Korean medical doctors who perform traditional Korean medicine (TKM) [6]. Reflecting this system, the number of clinicians in Korea provided by the Organization for Economic Cooperation and Development includes both of them [7].

There was a study on the use of CAM therapies in PD patients [8]. This study was conducted by Asan Medical Center in Seoul, Korea. It showed that 76% PD patients used one or more CAM therapies. This study reported that 57.6% used CAM therapies for improving motor symptoms, 19.6% for fatigue, 4.3% for pain, and 5.4% for constipation. However, this survey [8] was conducted in typical western medicine hospital. To the best of our knowledge, no study has been conducted by official medical institutions such as traditional Korean Medicine Hospital (KMH), which directly provide CAM therapies. Therefore, we retrospectively reviewed the medical records of patients who visited a KMH to identify the characteristics of PD patients who visited actual CAM clinics. In order to establish more suitable evidence and treatment strategies for PD patients who want to be treated with TKM or CAM therapies in the future, we investigated the characteristics of PD patients who visited a KMH, the reason for visiting KMH, and the contents of TKM therapies applied to the patients.

## 2. Materials and Methods

### 2.1. Subjects

The medical records of patients with the following criteria were selected for this study: (i) patients older than 19 years who were treated according to the International Standard Classification of Diseases and Causes of Death, PD (G20) or Parkinsonism (G22), and had their first visit at the Kyung Hee University KMH, Seoul, Korea from 1 January 2013 to 31 December 2017; (ii) patients whose medical records included demographic characteristics (sex, age, educational background, and occupation) and disease characteristics (disease durations, diagnosis, symptoms, and medication usage durations) on their initial visit day.

The following patients were excluded: (i) insufficient information in the medical record; (ii) patients initially treated with PD or Parkinsonism but finally diagnosed with other conditions.

### 2.2. Setting, Design, and Chart Review Methods

This retrospective chart review study was conducted based on the medical records of a single institution, Kyung Hee University KMH located in Seoul, Korea, and it was approved by the Institutional Review Board of Kyung Hee University KMH (KOMCIRB 2018-08-003). The following data were collected from medical records of each patient.

### 2.3. Demographic Characteristics

Sex, age, educational background, and occupation were investigated. Inpatients with missing information about educational background and occupation were queried. In the case of educational background, we classified it as uneducated, elementary school (6 years), middle school (9 years), high school (12 years), college (16 years), postgraduate graduate school (over 16 years), and nonresponse. Occupations were classified into tertiary industry, professionals, self-employed, primary industry, housework, unemployed, and others.

#### 2.3.1. Disease Characteristics

Based on the medical records, the following items were examined: PD or Parkinsonism diagnosis history before first visit, symptoms, age of onset, disease duration, duration of anti-Parkinsonian medication, surgery history (e.g., pallidectomy, thalamotomy, and DBS) and Hoehn and Yahr Stage (H-Y Stage) at the first visit.

Motor (including 4 major motor symptoms) and nonmotor symptoms were investigated. Among 4 major motor symptoms, postural instability and gait disturbance were investigated separately as these two symptoms could be related to the H-Y Stage index.

#### 2.3.2. Motivation for Visiting KMH

To determine the specific reasons for visiting the KMH in patients with PD and Parkinsonism, the following items were investigated: visits to medical institutions before the KMH and specific reasons for selecting TKM therapies.

If the patient had visited a KMH through other medical institutions, the medical institutions visited were divided into western medical institutions (hospitals and clinics) and TKM institutions (hospitals and clinics).

Specific reasons for the selecting TKM therapies were investigated based on the following two situations. If the patients had been diagnosed with PD or Parkinsonism already, they were categorized into the following six groups: to add TKM therapies, no improvement, newly developed symptoms, aggravation, side effects, and to stop anti-Parkinsonian medication. Patients who visited the KMH to add TKM therapies into the existing treatment without any special symptoms change were classified into the “to add on TKM therapies” group. Patients who had already taken anti-Parkinsonian medications after being diagnosed with PD or Parkinsonism but showed no improvement were classified into the “no improvement” group. In addition to existing symptoms during the course of treatment, patients who experienced other additional symptoms such as back pain, constipation, and insomnia were classified into the “newly developed symptoms” group. Patients who revealed deterioration of Parkinsonian symptoms were defined as the “aggravation” group. Patients with side effects of conventional therapies such as dizziness, dyspepsia, and vomiting were classified into the “side effects” group. The patients who wanted to stop their current anti-Parkinsonian medications and replace them with TKM therapies were classified into the “to stop anti-Parkinsonian medication” group.

The patients who were not diagnosed at the first visit in KMH were classified as follows: the group who wanted to receive medical examination for the current symptoms and TKM therapies without specific deterioration of symptoms and the group who visited due to recent aggravation of symptoms.

#### 2.3.3. Contents of TKM Therapies

The modality of TKM therapies (e.g., acupuncture, moxibustion, and herbal medicine) and inpatient treatment history were investigated.

### 2.4. Statistical Analysis

Overall, the descriptive statistics were used to present the ratio, average value, and standard deviation of each investigation. Comparison of the usage ratio of treatment modalities applied to inpatients and outpatients was performed by the chi-square test.

## 3. Results

**Figure 1:**
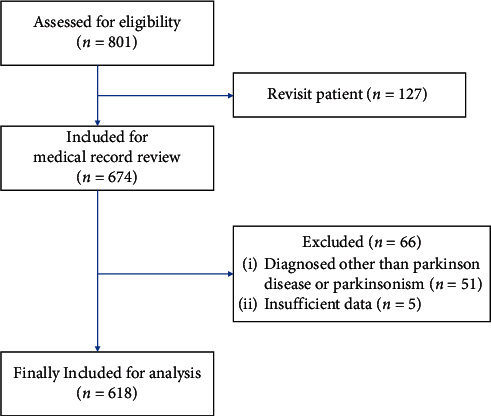
Flow diagram of the study.

A total of 801 patients who received TKM treatment at Kyung Hee University KMH from January 2013 to December 2017 were detected through a search of the medical records. Among them, 127 patients who had been already treated with TKM treatment were excluded. Additionally, 51 patients who had conditions other than PD, such as cerebral infarction, cerebral hemorrhage, and essential tremor, and 5 patients whose medical records were missing data were also excluded. Finally, the medical records of 618 patients were included in this review (see Figure 1).

### 3.1. Demographic Characteristics

Among 618 patients, 284 were men (46%), 334 were women (54%), and the mean age was 68.06 ± 9.80 (mean ± standard deviation) years (see Table 1).

According to the results of a survey of 129 inpatients with information on their educational background and occupation, the number of elementary school graduates (31.0%) was the highest, followed by high school graduates, middle school graduates, college graduates, uneducated, and postgraduate school graduates (see Table 1). Occupations were as follows: housework 54 (41.9%), followed by unemployed 40 (31.0%). Seven were tertiary industry workers (5.4%), six were primary industry workers (for example, agriculture, fisheries, livestock, and forestry) (4.7%), and four were professionals and self-employed (3.1%), respectively.

### 3.2. Disease Characteristics

Among 618 patients, 418 (67.6%) had already been diagnosed with PD or Parkinsonism before the KMH visit, while 200 (32.4%) had not been diagnosed until the first KMH visit. The age of onset (609 patients with information) was 65.46 ± 10.36 years, and the duration of the disease was 2.61 ± 2.95 years. The duration of the anti-Parkinsonian medication (324 patients with information) was 2.56 ± 2.94 years. The median Hoehn and Yahr Stage (617 patients) was Stage I. The number of patients for each Stage was Stage I, 314; Stage II, 213; Stage III, 75; Stage IV, 3; and Stage V, 12. The most common symptom was gait disturbance (56.6%). Tremor was seen in 54.4% patients, and 47.7% patients showed nonmotor symptoms, followed by bradykinesia, rigidity, and postural instability. Three patients underwent DBS related to PD; no patients had undergone any other brain surgeries (see Table 2).

### 3.3. Motivation for Visiting KMH

About 90% of the patients had visited other medical institutions before they visited to the KMH. Five hundred six patients (81.9%) visited the KMH via western medical institutions (hospital and clinic), and 67 (7.3%) visited via other TKM institutions (hospital and clinic). The remaining 67 patients (10.8%) visited without visiting other medical institutions.

There were 608 patients with a record of motivation for visiting KMH. Among patients diagnosed with PD or Parkinsonism before visiting the KMH, 136 (22.3%) visited to “add-on TKM therapies.” Ninety-seven patients (16.0%) visited due to “aggravation,” even though they were receiving western medical standard treatments and wanted to try adjunctive treatments. The reasons for visiting KMH were as follows: no improvement (73 patients, 12.0%), newly developed symptoms (45 patients, 7.4%), side effects of conventional therapies (43 patients, 7.1%), and to stop anti-Parkinsonian medication (22 patients, 3.6%).

**Figure 2:**
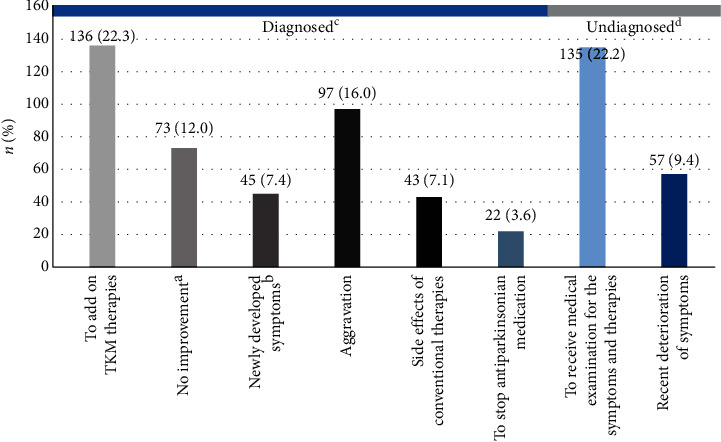
Motivations of seeking alternative therapies. ^a^No improvement: no change in symptoms after taking medicine or no response to levodopa and suspect other diseases; ^b^newly developed symptoms: new symptoms occur during treatment; ^c^diagnosed: diagnosed with idiopathic Parkinson disease or Parkinsonism when they visited the hospital; ^d^undiagnosed: not diagnosed when they visited the hospital.

Among patients who had not been diagnosed before visiting the KMH, 135 (22.2%) wanted to receive medical examination for the current symptoms and TKM therapies without specific deterioration of symptoms, followed by 57 (9.4%) who visited due to the recent deterioration of symptoms (see Figure 2).

### 3.4. Contents of TKM Therapies

**Figure 3:**
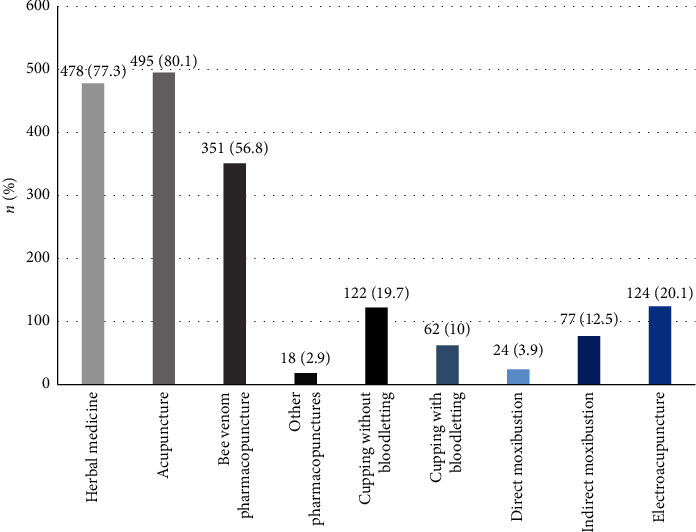
Usage details of TKM therapy regimen. TKM: traditional Korean medicine.

Inpatient treatment was performed on 129 (20.9%) patients. We examined all the TKM therapies administered to 618 patients. Acupuncture was the most used treatment, 495 patients (80.1%) and 478 patients (77.3%) were prescribed herbal medicines, and three hundred and fifty-one patients (56.8%) received bee venom pharmacopuncture therapy. The details of application of other treatments are as follows: electroacupuncture (124 patients, 20.1%), cupping without bloodletting (122 patients, 19.7%), indirect moxibustion (77 patients, 12.5%), cupping with bloodletting (62 patients, 10%), direct moxibustion (24 patients, 3.9%), and pharmacopunctures other than bee venom pharmacopuncture (18 patients, 2.9%). Seven patients received Qigong therapies (see Figure 3).

In the inpatient treatment, most treatment modalities were significantly more used than in the outpatient treatment. Only other types of pharmacopuncture showed no statistically significant difference between the two groups, but this also showed a high tendency of usage in inpatient treatment (inpatients vs outpatients: 75.2% vs 51.9%, *p*=0.238) (Table 3).

The most frequently prescribed herbal medicine (144 cases) was modified Ukgansan, followed by 99 cases of Uchashinkihwan, 89 cases of Jakyakgamchotang, 80 cases of Yukgunjatang, 53 cases of Bojungikgitang, 47 cases of Rhubarb capsule, 45 cases of Gamigwibitang, 39 cases of Geopungchunghyuldan, 36 cases of Danggwijakyaksan, and 31 cases of Majainhwan (see Table 4).

## 4. Discussion

Most medications currently used for PD aim to control motor symptoms and do not slow or reverse the natural progression of the disease. Levodopa, which is a typical medication for PD, is effective for bradykinesia and rigidity. However, in the chronic stage of PD, most patients develop motor complications associated with levodopa and might experience falls, dysphonia, autonomic neuropathy, depression, and dementia that do not respond properly to dopamine replacement therapy [1]. Furthermore, nonmotor symptoms accompanying PD, such as cognitive impairment, mood disorder, autonomic neuropathy, and sleep disorder, decrease patients' quality of life. Due to this, the characteristics of PD such as slow and long progression, widespread symptoms, and insufficient treatment to control motor and nonmotor symptoms lead to an increase in patients' interest in CAM therapies [9].

Previous studies [10–14] on CAM therapies such as TKM for PD mainly reported the therapeutic effects of CAM therapies through case reports or randomized controlled trials. However, to the best of our knowledge, no study has observed the characteristics of patients with PD or Parkinsonism who seek CAM therapies; that is, no study has been conducted to answer the question, “why are they looking for CAM therapies?” Two studies [8, 15] had been conducted to answer this question; however, they were conducted by an institution that does not implement CAM therapies. Therefore, in this study, we investigated the characteristics of patients visiting a KMH, which is the representative and official institution in Korea for CAM therapies. We investigated their motivation for seeking CAM therapies as well as the detailed treatment modalities of TKM by examining medical records.

A total of 618 patients' medical records were investigated. The average age of patients was 68.06 ± 9.80 years, similar to 65.7 years [15] and 63.0 ± 8.9 years [8], as previously found in studies conducted at Johns Hopkins and Asan Medical Center for the use of CAM in PD patients. According to the 2015 Korean education completion rate investigation [16], high school education completion rate for 55 to 64 years was 18%, which is significantly lower than 69% for 25 to 34 years. The patients in this study seem to have a high percentage of senior citizens, indicating a high percentage of elementary and middle school graduates. Moreover, considering the average age of retirement, the ratio of housework and unemployed people was high.

Before visiting the KMH, 67.6% of patients were already diagnosed with PD or Parkinsonism, compared with 32.4% who were not diagnosed until the time of first visit to the KMH. Of the total 418 patients who were already diagnosed, 324 (77.5%) had been treated with anti-Parkinsonian medications. The incidence age of patients in this study was higher than the average incidence age of 56.8 years (Johns Hopkins Hospital) [15] and 54.7 ± 11.6 years (Asan Medical Center) [8] seen in the previous study, which analyzed the characteristics of patients using CAM therapies. However, the average disease duration for PD in this study was shorter than 9.0 years (Johns Hopkins Hospital) [15] and 8.0 ± 5.3 years (Asan Medical Center) [8] seen in the previous study.

These results are likely to be explained as follows: patients who developed PD at a later age than before visited the hospital for CAM therapies within a short period. Regarding the fact that the number of Koreans using TKM was the highest (90.6%) among patients over 60 years in a survey on the current status of use of TKM in South Korea [17], it is thought that they were in an environment where they were easily exposed to TKM, such as the recommendation of their peers. The preference for CAM therapies, which are represented by TKM in Korea, was likely due to the higher accessibility to TKM than is seen in western countries. Moreover, the fact that the patients who visited KMHs are highly familiar with TKM might have resulted in a shorter duration of PD compared to that seen in previous studies. In addition, we could also assume that the recent interest in CAM therapies represented by traditional medicine may have affected this phenomenon. The Hoehn and Yahr Stage, which was lower than observed in previous studies [8, 15], also appears to be the result of relatively early visits to the KMH, which is in turn affected by the above factors.

More than half the patients complained of gait disturbance and tremor, while 47.7% complained of nonmotor symptoms that could be directly related to their quality of life. There were three patients who underwent DBS; no other surgeries were performed. This suggests that most PD patients usually visited the KMH before surgery was recommended or chosen. DBS is a surgical procedure performed when idiopathic PD is diagnosed or when a dopamine-acting drug shows a clear positive response and is not likely to be performed if the patient is over 75 years old or has a mental illness. According to previous reports, the mean disease duration before DBS was 13 years [18]. In this study, the mean disease duration was relatively short (2.61 ± 2.95 years). This is the likely reason for fewer patients who had undergone DBS.

Most patients (89.2%) in this study had been visiting other medical institutions before visiting the KMH. According to the “Survey on the Utilization of and the Consumption of Traditional Korean Medicine 2017” [17] conducted by the Korean Ministry of Health and Welfare, 50.4% of outpatients and 46.9% of inpatients in a TKM visited other medical institutions to treat the same symptoms before visiting TKM institutions. In a study in 2017 where a KMH conducted the survey, the ratio of first-aid medical institutions to first-aid TKM clinics was higher than that of first-aid medical institutions. Since this study was conducted at a KMH, which is a higher-level medical institution than the primary TKM clinic, it is assumed that the numbers of the patients who had visited other medical institutions before visiting TKM institution were higher than the results of the 2017 survey. Among the patients who had already been diagnosed with PD or Parkinsonism and visited KMH, the main motivation for patients visiting the KMH before being diagnosed with PD was that they wanted to receive traditional Korean medicine treatment along with investigation for current symptoms. The next frequent motivation was the recent worsening of the symptoms, for which no cause was apparent. Although 81.9% of patients visited via western medical institutions, only 67.6% had been diagnosed before visiting a traditional KMH. The reasons for this phenomenon were as follows: the previous medical institutions recommended visiting other hospitals as no special abnormalities were identified after performing brain imaging such as brain magnetic resonance imaging or recommended higher-level hospitals to conduct additional examinations.

Acupuncture was the most commonly used treatment (80.1%), 77.3% were prescribed herbal medicine, and 56.8% received bee venom pharmacopuncture treatment. In addition, patients received electroacupuncture, cupping without bloodletting, indirect moxibustion, cupping with bloodletting, direct moxibustion, and other types of pharmacopuncture besides bee venom. According to the “Survey on the Utilization of and the Consumption of TKM 2017” [17], 90.2% of patients received acupuncture treatments when using TKM, followed by cupping (53.0%), moxibustion (49.1%), and TKM physical therapy (40.2%). This result is similar to the treatment utilization rate in this study. The usage rate of treatment modalities (excluding other types of pharmacopuncture) was significantly higher in inpatients than in outpatients. This result suggests that more comprehensive TKM therapies had been applied in inpatients than in outpatients.

The most frequently prescribed herbal medicine was modified Ukgansan, which was prescribed a total of 144 times, followed by Uchashinkihwan (99 times), Jakyakgamchotang (89 times), Yukgunjatang (80 times), Bojungikgitang (53 times), Rhubarb capsule (47 times), Gamigwibitang (45 times), Geopungchunghyuldan (39 times), and Majainhwan (31 times). It has been confirmed that the most commonly used modified Ukgansan, Uchashinkihwan, and Jakyakgamchotang were mainly used to improve the motor symptoms of PD or Parkinsonism, such as tremor, bradykinesia, and rigidity, while other prescriptions were found to have been used mainly to improve nonmotor symptoms. The main purpose of each prescription is as follows: Rhubarb capsule and Majainhwan for constipation, Gamigwibitang for cognitive decline, Yukgunjatang for digestive disorders, and Bojungikgitang for systemic weakness.

There have been studies on the use of CAM for patients with PD; however, this study is the first one wherein the investigation was conducted directly by a KMH. It is also meaningful that it was targeted at medical service users who actually visited for CAM. Through this study, we could identify the characteristics and treatment status of patients who visited for CAM clinics.

There are some limitations in this study. First, because of the characteristics of retrospective chart review design, every demographic characteristic was not evaluated in all subjects. For example, duration of anti-Parkinsonian medication was investigated in only 324 of 618 subjects. Second, we conducted the present study based on the medical records of one KMH. Therefore, the results of the present study could not be interpreted as overall characteristics of PD patients who prefer TKM or CAM therapies. To overcome these limitations, it is necessary to conduct a prospective, multicenter study using questionnaires based on the result of the present study. A different questionnaire for survey depending on whether patients are diagnosed with PD will have to be required to obtain the necessary information. Further investigation should be conducted on the contents such as academic background, job information, and surgical recommendations that were not investigated in this study. In addition, in this study, the effect of CAM therapies was not evaluated, because each patient had a different treatment period and each patient had a limited time to assess symptom progress. Therefore, any further study should be conducted as a prospective case series.

## Figures and Tables

**Table 1:** Demographic characteristics of patients.

Characteristics	Total numbers of patients	Results
Sex (male/female), *n* (%)	618	284/334 (46/54)

Age, yr, mean ± standard deviation	618	68.06 ± 9.80

Educational background	219	
Uneducated, *n* (%)		10 (7.8)
Elementary school graduate, *n* (%)		40 (31.0%)
Middle school graduate, *n* (%)		18 (14.0)
High school graduate, *n* (%)		23 (17.8)
College graduate, *n* (%)		16 (12.4)
Postgraduate school, *n* (%)		6 (4.6)
Nonresponse, *n* (%)		16 (12.4)

Occupations	219	
Tertiary industry, *n* (%)		7 (5.4)
Professionals, *n* (%)		4 (3.1)
Self-employed, *n* (%)		4 (3.1)
Primary industry, *n* (%)		6 (4.7)
Housework, *n* (%)		54 (41.9)
Unemployed, *n* (%)		40 (31.0)
Others, *n* (%)		14 (10.8)

**Table 2:** Clinical characteristics of patients.

Characteristics	Total numbers of patients	Results
Diagnosed/undiagnosed^†^, *n* (%)	618	418/200 (67.6/32.4)

Age of onset (yrs)	609	65.46 ± 10.36

Disease duration (yrs)	609	2.61 ± 2.95

Duration of anti-Parkinsonian medication (yrs)	324	2.56 ± 2.94

Surgery history, *n* (%)	618	3 (0.5)
Deep brain stimulation	618	3 (0.5)
Others (pallidectomy and thalamotomy)	618	0 (0)

Hoehn and Yahr Stage	617	1^‡^
Stage I, *n* (%)		314 (50.9)
Stage II, *n* (%)		213 (34.5)
Stage III, *n* (%)		75 (12.2)
Stage IV, *n* (%)		3 (0.5)
Stage V, *n* (%)		12 (1.9)

Major symptoms^*∗*^	618	
Tremor		336 (54.4)
Bradykinesia		277 (44.8)
Rigidity		143 (23.1)
Postural instability		73 (11.8)
Gait disturbance		349 (56.5)
Nonmotor symptoms		295 (47.7)

Values are mean ± SD or number (%). ^†^Whether to diagnose at the time of initial stage. ^‡^Median value. ^*∗*^Overlapped symptoms included.

**Table 3:** Differences in TKM therapy regimen between inpatients and outpatients.

TKM therapy modality	Inpatients (*n* = 129)	Outpatients (*n* = 489)	*p* value
Herbal medicine, *n* (%)	129 (100.0)	349 (71.4)	<0.001^*∗*^

Acupuncture, *n* (%)	129 (100.0)	366 (74.8)	<0.001^*∗*^

Bee venom pharmacopuncture, *n* (%)	97 (75.2)	254 (51.9)	<0.001^*∗*^

Other pharmacopuncture, *n* (%)	2 (1.6)	16 (3.3)	0.238

Cupping			
Without bloodletting, *n* (%)	106 (82.2)	16 (3.3)	<0.001^*∗*^
With bloodletting, *n* (%)	39 (30.2)	23 (4.7)	<0.001^*∗*^

Moxibustion			
Direct, *n* (%)	21 (16.3)	3 (0.6)	<0.001^*∗*^
Indirect, *n* (%)	64 (49.6)	13 (2.7)	<0.001^*∗*^

Electroacupuncture, *n* (%)	93 (72.1)	31 (6.3)	<0.001^*∗*^

Values are number (%). TKM; traditional Korean medicine. *p* values were calculated by the chi-square test. ^*∗*^*p* value <0.05.

**Table 4:** Use of herbal medicines.

Frequency of use	Name of herbal medicine (Chinese characters' name and frequency)
More than 30 times	Modified Ukgansan (抑肝散加味, 144), Uchashinkihwan (牛車腎気丸, 99), Jakyakgamchotang (芍藥甘草湯, 89), Yukgunjatang (六君子湯, 80), Bojungikgitang (補中益氣湯, 53), Rhubarb capsule (大黃, 47), Gamigwibitang (加味歸脾湯, 45), Geopungchunghyuldan (祛風清血丹, 39), Danggwijakyaksan (當歸芍藥散, 36), Majainhwan (麻子仁丸, 31)

More than 20 times	Gyejigachulbutang (桂枝加朮附湯, 28), Gyejigayonggolmoryeotang (桂枝加龍骨牡蠣湯, 28), Bosimsahwachunggantang (補心瀉火淸肝湯, 25), Gyejibongnyeonghwan (桂枝茯苓丸, 22), Banhabaekchulcheonmatang (半夏白朮天麻湯, 20), Boikyangwitang (補益養胃湯, 20), Oryeongsan (五苓散, 20)

More than 10 times	Gamisoyosan (加味逍遙散, 19), Yungmijihwangtang (六味地黃湯, 18), Banhahubaktang (半夏厚朴湯, 17), Bosimdan (補心丹, 17), Banggihwanggitang (防己黃芪湯, 15), Chunghyeoldan (淸血丹, 15), Maengmundongtang (麥門冬湯, 13), Banhasasimtang (半夏瀉心湯, 13), Sihogyejitang (柴胡桂枝湯, 13), Ikgibohyeoltang (益氣補血湯, 13), Gujeongjihwanghwan (求精地黃丸, 12), Cheongeumjowitang (千金調胃湯, 12), Woohwangcheongshimwon (牛黃淸心元, 10), Cheonginyukwaehwan (淸咽癒快丸, 10), Hwangnyeonhaedoktang (黃連解毒湯, 10)

9 times	Sopungsungiwon (疎風順氣元), Ojeoksan (五積散), Jaeumganghwatang (滋陰降火湯), Jodeungsan (釣藤散)

8 times	Sihogayonggolmoryeotang (柴胡加龍骨牡蠣湯)

7 times	Geumgonghwan (金蚣丸), Yanggyeoksanhwatang (凉膈散火湯), Jayuntang (滋潤湯), Gammaekdaejotang (甘麥大棗湯), Chilmulganghatang (七物降下湯)

6 times	Seunggitanggami (承氣湯加味), Ijungtang (理中湯), Ijintang (二陳湯)

5 times	Daesihotanggami (大柴胡湯加味), Danggwisayeokgaoshuyusaenggangtang (當歸四逆加吳茱萸生薑湯), Socheongnyongtang (小靑龍湯), Ssanghwatanggami (雙和湯加味), Insukbosimtang (仁熟補心湯), Cheongsimyeonjatang (淸心蓮子湯)

4 times	Gwakjeongtanggami (藿正湯加味), Bosimgeonbitang (補心健脾湯), Saengmaeksan (生脈散), Siryeongtang (柴苓湯), Cardiotonic Pills (丹蔘滴丸), Jeoryeongtang (豬苓湯)

3 times	Geonbihwan-Granule (健脾丸顆粒), Gwiinansimtang (歸仁安心湯), Danggwisayeokgaosuyusaenggangtang (當歸四逆加吳茱萸生薑湯), Daeseunggitang (大承氣湯), Bojeongdan (補精丹), Ansimsan (安心散), Uiiintang (薏苡仁湯), Jakgamhwangsinbutang (芍甘黃辛附湯), Cheongjang (淸腸)-Capsule, Hyeongbangjihwangtang (荊防地黃湯)

2 times	Galgeuntang (葛根湯), Gochimmuusan (高枕無憂散), Gongjindan (供辰丹), Gilchogeundan (吉草根丹), Bohyeolansintang (補血安神湯), Saengjin (生津), Sopunghwalhyeoltang (疎風活血湯), Sohwageonjungtang (消化健中湯), Ansimondamtang (安心溫膽湯), Oncheonghyeol Granule (溫淸血顆粒), Wongi-jelly (元氣-jelly), Cheongsimjihwangtang (淸心地黃湯), Cheonmagudeungeum (天麻鉤藤飮), Palmultang (八物湯)

1 time	Gagamyanggyeoksan (加減凉膈散), Galgeunhaegitang (葛根解肌湯), Geojeokchwisan (祛積聚散), Gyeontongdodamtang (蠲痛導痰湯), Naesohwajungtang (內消和中湯), Noebotang (腦補湯), Danggwiyukhwangtang (當歸六黃湯), Daegeonjungtang (大建中湯), Daebangpungtang (大防風湯), Daebowonjeon (大補元煎), Doinseunggitang (桃仁承氣湯), Dojeokganggitang (導赤降氣湯), Bangpunghaedoktang (防風解毒湯), Baekjunghwan (百中丸), Bosingeonyangtang (補腎健陽湯), Sagunjatang (四君子湯), Sayuktanggami (四六湯加味), Samgieum (三氣飮), Samryeongbaekchulsan (蔘苓白朮散), Samchulgeonbitang (蔘朮健脾湯), Saengjisan (生地散), Seogyeongtang (舒經湯), Seungmagalgeuntang (升麻葛根湯), Seungyangikgibujatang (升陽益氣附子湯), Singihwan (腎氣丸), Sipjeondaebotang (十全大補湯), Yeoldahansotang (熱多寒少湯), Yeonggyechulgamtang (苓桂朮甘湯), Yongnoesohabwon (龍腦蘇合元), Insampaedoksan (人蔘敗毒散), Jaeumyangyeongtang (滋陰養榮湯), Jowitanggami (調胃湯加味), Jinmutang (眞武湯), Cheongsangbohatang (淸上補下湯), Cheongsimganghwawon (淸心降火元), Cheongsimtanggami (淸心湯加味), Cheongwisagansan (淸胃瀉肝散), Cheongpyesagantang (淸肺瀉肝湯), Cheonghyeolganggitang (淸血降氣湯), Paegamsan (貝甘散), Hachulbosimtang (夏朮補心湯), Hyangsayangwitang (香砂養胃湯), Hyeolbuchugeotang (血府逐瘀湯), Hyeongbangsabaeksan (荊防瀉白散), Hwanggigeonjungtang (黃芪建中湯), Hoesusan (回首散)

## Data Availability

The data used to support the findings of this study are included within the article.
